# Hydrogen peroxide as an auxiliary treatment for COVID-19 in Brazil: a randomized double-blind clinical trial

**DOI:** 10.4178/epih.e2021051

**Published:** 2021-08-03

**Authors:** Marielle Bazzo Di Domênico, Kauê Collares, Renan Brandenburg dos Santos, Ulysses Lenz, Vinícius Picoli Antunes, Vinicius Webber Godinho, Henrique Cesca, Thales Henrique Jincziwski Ponciano, Pedro Henrique Corazza

**Affiliations:** Graduate Program in Dentistry, Dental School, University of Passo Fundo, Passo Fundo, Brazil

**Keywords:** Hydrogen peroxide, COVID-19, SARS-CoV-2, Brazil

## Abstract

**OBJECTIVES:**

This study evaluated the effectiveness of hydrogen peroxide (H_2_O_2_) as mouthwash and nasal spray on symptom relief in coronavirus disease 2019 (COVID-19) patients.

**METHODS:**

Patients positive for severe acute respiratory syndrome coronavirus 2 (SARS-CoV-2), who were treated in a hospital or at home, and patients’ family members (not positive for SARS-CoV-2), were randomized into 2 groups: experimental (1% H_2_O_2_ for gargling, 0.5% H_2_O_2_ for nasal wash), and control. Patients gargled the solution 3 times a day, and applied the nasal spray twice a day, for a 7-day period. Family members received the same treatment as the treated COVID-19 patient. The researchers contacted patients every 2 days over an 8-day period. An average post-treatment interval of 8 days passed before testing family members.

**RESULTS:**

The most frequent symptoms on day 0 were cough, loss of taste, and hyposmia; there were no significant differences between groups, independent of the period. The symptom of dyspnea presented a significant difference between days 2 and 4 (p<0.05). Among family members, 86.0% had no antibodies, 2.3% had antibodies, and 11.6% had active infections (4 in the experimental group and 6 in the control group). The most frequent adverse effects in the H_2_O_2_ group were a burning throat and nose.

**CONCLUSIONS:**

H_2_O_2_ was not effective for the relief of COVID-19 symptoms and was associated with reports of transient adverse effects.

## INTRODUCTION

After more than a year since its emergence in the city of Wuhan, China, severe acute respiratory syndrome coronavirus 2 (SARS-CoV-2) continues to kill thousands of people around the world. In recent months, many countries have faced a second—or even a third—wave of coronavirus disease 2019 (COVID-19) [1,2], which has been a major concern for authorities. SARS-CoV-2 shows a high capacity for transmission between individuals, through both direct (coughing, sneezing and inhaling saliva droplets) and indirect (contact with contaminated surfaces) routes [3], and it does not trigger any symptoms in many cases (up to 79% of cases) [4]. The median incubation period of SARS-CoV-2 is estimated to be 5 days (between 2 and 7 days), and 97.5% of patients who develop symptoms will do so within 11.5 days of infection [5,6]. The oropharynx and nasopharynx are closely related to disease transmission and evolution [7,8]. Zou et al. [8] analyzed the viral load in samples obtained from the nose and throat of symptomatic and asymptomatic patients. Higher viral loads were detected soon after the onset of symptoms, and the loads were higher in the nose than in the throat. According to Herrera et al. [9], the oral viral load of SARS-CoV-2 was associated with the severity of COVID-19, implying that a reduction in the oral viral load could be associated with a decrease in disease severity [10]. Similarly, a decrease in the oral viral load would decrease the amount of virus expelled and reduce the risk of transmission, which is high between individuals from the same family group [11].

Hydrogen peroxide (H_2_O_2_) at low concentrations has been used over the years for several purposes. It disrupts the lipid membranes of some viruses through the action of oxygen free radicals. Studies have reported that coronavirus 229E and other enveloped viruses can be inactivated at H_2_O_2_ concentrations of around 0.5% [12,13]. Even without scientific evidence in humans, many regulatory commissions around the world suggested using H_2_O_2_ before dental care, based exclusively on previous findings of in vitro studies.

Thus, the purpose of the present study was to evaluate the effectiveness of hydrogen peroxide in the form of mouthwash (1.0%) and nasal spray (0.5%) as an auxiliary treatment for COVID-19 patients. The hypotheses were that the treatment would be effective at relieving symptoms related to the disease and reducing infection in patients’ family members. This brief communication supplements the already published preliminary data [14] of the study by providing the final results.

## MATERIALS AND METHODS

### Study design

This study was a randomized, double-blind, placebo-controlled clinical trial to assess the effectiveness of gargling and nasal wash with H_2_O_2_ to reduce COVID-19 symptoms in adults and transmission between family members.

**Figure 1.**
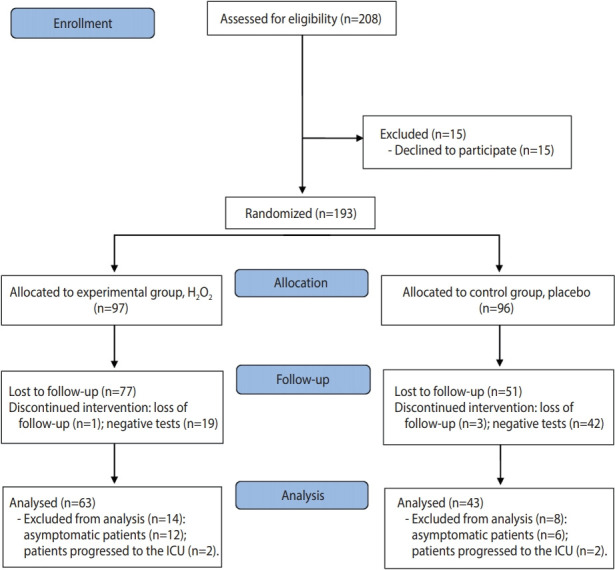
CONSORT (Consolidated Standards of Reporting Trials) 2010 flow diagram. H_2_O_2_, hydrogen peroxide; ICU, intensive care unit.

The study was registered in the Brazilian Registry of Clinical Trials (registration No. RBR-6sx3sz) and followed the CONSORT (Consolidated Standards of Reporting Trials) criteria for clinical studies (http://www.consort-statement.org/). The CONSORT flow diagram is presented in Figure 1.

### Patients

Eligible patients (n= 208) were men and women with a reverse-transcription polymerase chain reaction (RT-PCR) examination positive for SARS-CoV-2, who were residents or under treatment in a city in southern Brazil. Patients were treated in hospital beds or at home (in isolation). During the research period (July to November 2020), there were 9,822 confirmed cases of COVID-19 in the city of Passo Fundo, where the study was conducted.

The eligibility criteria were as follows: testing positive for SARS-CoV-2 and receiving the diagnosis less than 3 days before the intervention or waiting for the test result; being hospitalized outside the intensive care unit or in isolation at home; having the physical capacity to gargle and apply the nasal spray on one’s own; presenting moderate or mild COVID-19 symptoms; and agreeing to participate in the study.

Patients waiting for their test results who were initially included in the study, but who tested negative for SARS-CoV-2, were excluded after diagnosis (19 in the experimental group and 42 in the control group). Patients’ family members who had never tested positive for SARS-CoV-2 before the experiment were also included in the study (n= 97); family members who had already tested positive were not included.

Details about the randomization, blinding procedure, researcher team training, and preparation of the solutions can be obtained in the preliminary original article [14].

### Interventions

The 2 study groups were:

Experimental (n= 97): 1% H_2_O_2_ for gargling, and 0.5% H_2_O_2_ for a nasal wash, in which patients gargled with a solution composed of 1% H_2_O_2_ and mint essence for 30 seconds, 3 times a day, for a 7-day period. One dose of the nasal spray was applied to each nostril, twice a day, for a 7-day period. The nasal solution was composed of 0.5% H_2_O_2_ and mint essence.
Control (n= 96) (placebo): The control group gargled and applied the nasal spray in the same manner described for the experimental group. The placebo solution was composed of distilled water and mint essence.

Patients’ family members received the same treatment as their treated family member. Each participant had his or her own kit. Each family member included in the research signed a voluntary informed consent term, as did the index patient.

### Data collection

On day 0 (first contact), the patient was invited to participate in the study, and a kit composed of the mouthwash and nasal spray was provided, according to randomization. On the same day, individual variables were obtained from a questionnaire developed by the researchers. Socioeconomic and socio-demographic characteristics, comorbidities and the patient’s symptoms at baseline were recorded. Hospitalized patients were monitored in the hospital every 2 days, over an 8-day period, by 2 trained researchers (a total of 4 visits). If the patient was discharged before the end of the survey, follow-up was performed by phone. The patients treated at home were contacted by the same researchers every 2-day, over an 8-day period; this contact was made by phone. The symptoms of fever, cough, hyposmia, loss of taste, dyspnea, and sore throat were evaluated.

### Outcomes

#### Patients

During follow-up, patients were asked about their symptoms with the question: “Do you have any of these symptoms? Fever, cough, hyposmia, loss of taste, dyspnea, sore throat?” If they did, they were asked about the severity of the symptom (1, mild; 2, moderate; or 3, severe). The possible adverse effects of the solution were also recorded with the question: “Did you have any of these symptoms after using the solution? A burning sensation in your mouth, a burning sensation in your throat, food tasting unpleasant after use, the feeling of having a thick tongue, a burning sensation in your nose?” If they did, they were asked about the severity of the symptom (1, mild; 2, moderate; or 3, severe). Clinical relief of symptoms was defined as a reduction in the previously reported value (1, 2, or 3) between days 0-2, 2-4, or 4-6.

The patient’s self-reported information was recorded on the same form as other clinical data. All data were converted to an electronic database.

#### Family members

The primary outcome assessed in patients’ family members was “infected” or “not infected.” After 7 days of using the solutions, an average interval of 8 days passed [15] before family members were tested. A blood sample test was used (COVID-19 IgG/IgM test, DFL & Humasis Co., Anyang, Korea). Four trained researchers applied the tests, in accordance with the manufacturer’s instructions. They did not know which group the individual belonged to.

### Sample calculation

The primary outcome of this study was the absolute risk of symptom reduction 8 days after a COVID-19 diagnosis. For the sample calculation, the absolute risks were obtained through a pilot study with 14 patients, where 73% of the individuals in the intervention group and 33% in the placebo group showed a reduction in COVID-19 symptoms after 8 days. To be able to detect this 40% difference between the placebo and the reference group, a total of 30 patients per group was required to achieve 80% power with a 5% bilateral significance.

### Statistical analysis

Stata version 14 (StataCorp., College Station, TX, USA) was used for data analysis. A descriptive analysis was initially performed to determine the relative and absolute frequency of patient characteristics. The rate of individuals who showed relief of symptoms during days 0-2, 2-4, and 4-6 were compared using the Fisher exact test (α= 0.05). The results of the tests on family members were tabulated, and the relative frequency was calculated. The adverse effects of the solutions were compared using the Fisher exact test (α= 0.05). Efficacy analysis was performed on an intention-to-treat basis, including all the patients who had undergone randomization. Hazard ratios (HRs) with 95% confidence intervals (CIs) were calculated using a Cox proportional-hazards model.

### Ethics statement

Ethical approval was obtained from the National Research Ethics Commission (CONEP, #4.071.153) and from Hospital das Clinicas, the hospital involved in the research, in the city of Passo Fundo, Brazil. All patients (or their legal guardians) approved and signed the informed consent form.

## RESULTS

Of the 208 patients assessed for eligibility, 15 declined to participate in the research and 193 patients were randomized, with 97 allocated to the experimental group and 96 to the control group. In the experimental group, 1 patient was lost to follow-up and 19 patients were excluded due to having negative RT-PCR results; in the control group, 3 patients were lost to follow-up and 42 patients were excluded because they had negative RT-PCR results (Figure 1). In this study, 106 patients were evaluated after accounting for patients who were lost to follow-up or excluded; of these, 35 were hospitalized and 71 were in treatment at home. The characteristics of the participants are shown in Table 1.

The most frequent symptoms on day 0 were cough (52.4% in the experimental group, 62.8% in the control group), loss of taste (44.4% in the experimental group, 48.8% in the control group), and hyposmia (41.3% in the experimental group, 46.5% in the control group) (Table 2). All symptoms showed some relief during the 8 days of follow-up.

Table 2 illustrates symptom relief throughout the treatments and presents a statistical comparison of the groups. The symptoms of cough, loss of taste, hyposmia, sore throat and fever did not show statistically significant differences between both groups, independent of the evaluated period. Dyspnea presented statistical significance between days 2 and 4 (p< 0.05), as 92.3% of the patients in the control group had relief, whereas symptom relief for dyspnea was reported by 58.3% of the experimental group. Patients treated with H_2_O_2_ did not present a significantly different time to clinical relief compared to the control group in the intention-to-treat population (HR for clinical relief, 0.99; 95% CI, 0.60 to 1.63).

Forty-one patients (38.7%), including 10 hospitalized patients and 31 patients receiving treatment at home, had at least 1 person living in the same house who was infected before the study began. There were 170 family members living in the same residence as the patients included in the study, 73 of whom had already tested positive and were not included in the study. Thus, 97 family members were included: 61 in the experimental group and 36 in the control group. Of these, 9 tested positive before completing the study (3.0± 1.2 days; 1 in the control group and 8 in the experimental group). Eighty-six family members were tested, on average 7.44 ± 2.44 days after the end of the treatment. Of these family members, 74 (86.0%) tested negative, 2 (2.3%) were immunoglobulin G (IgG)-reactive, 2 (2.3%) were immunoglobulin. M (IgM)-reactive, and 8 (9.3%) were IgG-reactive and IgM-reactive (Table 3).

The symptoms most frequently reported by the participants who used the H_2_O_2_ solution were burning throat (22.2%) and burning nose (31.7%) on day 2 (Table 4). Both symptoms showed a statistically significant difference between the groups for the 3 days that were evaluated.

## DISCUSSION

Initially, 193 patients were randomized in the present study. Subsequently, 4 were lost to follow-up, 4 progressed to the intensive care unit, 61 tested negative for COVID-19, and 18 were excluded from the statistical analysis because they were asymptomatic on day 0. The final study sample consisted of 106 symptomatic adults, hospitalized or in treatment at home for COVID-19. The randomization in the present study was stratified, considering the characteristics “hospitalized” and “in treatment at home.” Most patients were between 36 years and 59 years old (56.6%), and hypertension (22.6%) and diabetes (11.3%) were the most frequent comorbidities. The results were similar to other studies in the area, where the average age of infected patients was 49 years old [16], and the most prevalent comorbidities were the same. These comorbidities are also associated with disease severity and prognosis [17,18].

In the present study, the mean time between the onset of symptoms and the beginning of the use of the solutions (9.2± 3.4 days) is a likely explanation of the lower frequency of fever than reported elsewhere in the literature. It is known and determined by the World Health Organization that RT-PCR testing must be performed 3-7 days after the first symptoms [19]. However, the local public health system was taking 7-15 days from the first symptoms to return the results, at which point many patients had no symptoms (and were excluded) or no longer had fever. In the present study, all symptoms showed some relief during the 8-day follow-up in both groups.

The data obtained in the present study demonstrated that the use of H_2_O_2_ as mouthwash and nasal spray was not effective on symptom relief in patients with COVID-19, rejecting the first study hypothesis. This hypothesis was formulated considering that H_2_O_2_ can damage or destroy the virus lipid layer, which could reduce the viral load of infected individuals and affect the symptoms of the disease [10,20]. The symptoms of cough, loss of taste, hyposmia, sore throat, and fever showed no significant differences between the H_2_O_2_ and placebo groups in any of the periods evaluated. Only dyspnea resulted in significance between the second and fourth days, in favor of the control group (p< 0.05). However, this difference cannot be attributed to the solutions, because between day 0 and day 2, a contrary effect was observed: 50.0% of patients from the H_2_O_2_ group and 25.0% of patients from the control group experienced relief. A recent study [21] testing the association between 1% H_2_O_2_ mouthwash and the viral load of patients with COVID-19 found no efficacy of the H_2_O_2_ solution in reducing the viral load. However, the results of this mentioned study could be considered inconclusive, due to the very small sample size (12 SARS-CoV-2-positive patients). The preliminary results of the present study already demonstrated no significant difference between the placebo and experimental groups for hospitalized patients. However, 75% of the experimental group patients presented a decrease in the symptom of difficulty breathing between days 0-2 [14].

Human-to-human transmission of SARS-CoV-2 occurs mainly between family members, including asymptomatic patients [5], and especially when a large number of people are living in the same residence. Data on SARS-CoV-2 transmission to family contacts is still limited. Patients’ family members who had never tested positive for SARS-CoV-2 before the experiment also used the solutions, and this study evaluated whether they became infected. There were 170 family members living in the same households as the included COVID-19 patients, 73 of whom had already been infected with SARS-CoV-2 and were excluded, leaving 97 family members who were included, 9 of whom had a positive diagnosis before finishing the 7-day use of the solutions and were also excluded. Eighty-six family members were tested after the end of the study using the rapid blood sample test. This test detects IgG and IgM, with a mean clinical sensitivity above 90% for a positive reaction after more than 4 days. The transmission between the index patients and the family members tested was lower in both groups than has been reported in the literature [22,23]. Due to the small number of positive family members, the second hypothesis regarding the infection of patients’ family members remains inconclusive. It is important to mention here the present randomized clinical trial was developed before the first reports of the Brazilian SARS-CoV-2 variant P.1 [24].

H_2_O_2_ has been used in dentistry for more than 70 years. H_2_O_2_ at 3% or less has been used daily for up to 6 years and has shown occasional transient irritating effects only in a small number of individuals with pre-existing ulceration, or when high levels of salt solutions have been simultaneously administered [25]. Even though the present study only prescribed the solutions for 7 days (gargling 3 times a day, nasal spray twice a day), some adverse effects were observed. The most frequent reports in the experimental group were burning throat and burning nose, resulting in statistically significant differences on all days of follow-up. Given the adverse effects, even though they were transient, and the lack of effectiveness, we advise against the use of H_2_O_2_ as a mouthwash and nasal spray to relieve COVID-19 symptoms and transmission, even at different concentrations or for different periods. The present study has some limitations. Positive patients showed some resistance to participating in the study, especially those who were hospitalized. In addition, the time between the first symptoms and informing the individual of their positive result meant that many patients no longer had symptoms when they were contacted to start the treatment. Seeking to circumvent this issue, the researchers included some patients even before their RT-PCR test results were known. However, approximately 80% of them tested negative and were eliminated from the study, which explains the difference in the sample size of the groups.

In conclusion, H_2_O_2_ was not effective for the relief of symptoms of patients with COVID-19. Moreover, it was associated with transient adverse effects such as burning sensations in the nose and throat.

## Figures and Tables

**Table 1.** Demographic characteristics of patients at baseline

Characteristics		Total	Experimental	Placebo	p-value
Total		106 (100)	63 (59.4)	43 (40.6)	
Sex					0.20
	Female	64 (60.4)	34 (54.7)	29 (45.3)	
	Male	42 (39.6)	38 (66.7)	14 (33.3)	
Age (yr)					0.40
	≤35	28 (26.4)	17 (60.7)	11(39.3)	
	36-59	60 (56.6)	33 (55.0)	27 (45.0)	
	≥60	18 (17.0)	13 (72.2)	5 (27.8)	
Skin color					0.10
	White	90 (84.9)	56 (62.2)	34 (37.8)	
	Non-White	16 (15.1)	7 (43.7)	9 (56.3)	
Education level					0.70
	Completed high school	76 (71.7)	46 (60.5)	30 (39.5)	
	University education (complete/incomplete)	30 (28.3)	17 (56.7)	13 (43.3)	
Family income (Brazilian real)					0.20
	≤3,162.00	54 (50.9)	29 (53.7)	25 (46.3)	
	>3,162.00	52 (49.1)	34 (65.4)	18 (34.6)	
No. of people in the same residence					0.50
	None	11 (10.4)	6 (54.5)	5 (45.5)	
	1	33 (31.1)	18 (54.5)	15 (45.5)	
	2	29 (27.4)	21 (74.4)	8 (27.6)	
	3	14 (13.2)	7 (50.0)	7 (50.0)	
	≥4	19 (17.9)	11 (57.9)	8 (42.1)	
People who tested positive in the same residence					0.01
	None	60 (56.6)	30 (50.0)	30 (50.0)	
	1	24 (22.6)	15 (62.5)	9 (37.5)	
	≥2	17 (16.0)	15 (88.2)	2 (11.8)	
Comorbidities					
	Cardiac	4 (3.8)	3 (75.0)	1 (25.0)	0.50
	Respiratory	6 (5.7)	1 (16.7)	5 (83.3)	0.02
	Diabetes	12 (11.3)	6 (50.0)	6 (50.0)	0.40
	Hypertension	24 (22.6)	13 (54.2)	11 (45.8)	0.50

Values are presented as number (%).

**Table 2.** Symptomatic individuals on day 0 who reported relief of symptoms for cough, loss of taste, hyposmia, dyspnea, sore throat, and fever over the first 6 days

Symptom	Total with symptom on day 0		Total patients with relief of symptoms on days 0-2			Total patients with relief of symptoms on days 2-4			Total patients with relief of symptoms on days 4-6		
	H_2_O_2_	Control	H_2_O_2_	Control	p-value	H_2_O_2_	Control	p-value	H_2_O_2_	Control	p-value
Cough	33 (52.4)	27 (62.8)	12/33 (36.4)	12/27 (44.4)	0.4	18/33 (54.5)	11/27 (40.7)	0.5	9/33 (27.3)	6/27 (22.2)	0.8
Loss of taste	28 (44.4)	21 (48.8)	10/28 (35.7)	6/21 (28.6)	0.7	13/28 (46.4)	13/21 (61.9)	0.1	10/28 (35.7)	3/21 (14.3)	0.1
Hyposmia	26 (41.3)	20 (46.5)	6/26 (23.1)	7/20 (35.0)	0.3	15/26 (57.7)	9/20 (45.0)	0.7	9/26 (34.6)	4/20 (20.0)	0.2
Dyspnea	16 (25.4)	16 (37.2)	8/16 (50.0)	4/16 (25.0)	0.1	7/16 (43.7)	12/16 (75.0)	<0.05	5/16 (31.3)	2/16 (12.5)	0.8
Sore throat	13 (20.6)	10 (23.3)	10/13 (76.9)	5/10 (50.0)	0.2	4/13 (30.8)	6/10 (60.0)	0.4	2/13 (15.4)	3/10 (30.0)	0.8
Fever	7 (11.1)	4 (9.3)	4/7 (57.1)	3/4 (75.0)	0.5	2/7 (28.6)	1/4 (25.0)	0.3	2/7 (28.6)	-	-

Values are presented as number (%). H_2_O_2_, hydrogen peroxide.

**Table 3.** Results of tests on family members

Variables	Total (n=86)	H_2_O_2_ (n=51)	Control (n=35)
Negative	74 (86.0)	45 (88.2)	29 (82.9)
IgG	2 (2.3)	2 (3.9)	0 (0.0)
IgM	2 (2.3)	1 (2.0)	1 (2.9)
IgG and IgM	8 (9.3)	3 (5.9)	5 (14.3)

Values are presented as number (%). IgG, immunoglobulin G; IgM, immunoglobulin M.

**Table 4.** Frequency of adverse effects on each day

Adverse effects	Day 2			Day 4			Day 6		
	H_2_O_2_	Control	p-value	H_2_O_2_	Control	p-value	H_2_O_2_	Control	p-value
Burning mouth	5 (7.9)	-	0.06	4 (6.6)	1 (2.3)	0.30	4 (6.7)	-	0.08
Burning throat	14 (22.2)	2 (4.8)	0.01	11 (18.0)	2 (4.8)	0.04	12 (20.0)	-	<0.05
Unpleasant taste of food after use	3 (4.8)	1 (2.3)	0.50	4 (6.6)	1 (2.3)	0.30	3 (5.0)	1 (2.3)	0.50
Feeling of thick tongue	-	-		3 (4.9)	-	0.10	1 (1.7)	-	0.40
Perceptible change in mucosa	-	-		-	-		-	-	
Burning nose	20 (31.7)	2 (4.8)	<0.01	21 (34.4)	2 (4.8)	<0.01	20 (33.3)	1 (2.3)	<0.01

Values are presented as number (%). H_2_O_2_, hydrogen peroxide.
